# Can ten days of heat acclimation training improve temperate-condition rowing performance in national-level rowers?

**DOI:** 10.1371/journal.pone.0273909

**Published:** 2022-09-01

**Authors:** Calvin P. Philp, Nathan W. Pitchford, Denis C. Visentin, Cecilia M. Kitic, James W. Fell, Martin Buchheit, Christopher T. Minson, John R. Gregory, Greig Watson

**Affiliations:** 1 Sport Performance Optimisation Research Team, School of Health Sciences, University of Tasmania, Launceston, Tasmania, Australia; 2 School of Health, Medical and Applied Sciences, Central Queensland University, Brisbane, Queensland, Australia; 3 French National Institute of Sport (INSEP), Laboratory of Sport, Expertise and Performance (EA 7370), Paris, France; 4 Institute for Health & Sport, Victoria University, Melbourne, VIC, Australia; 5 HIIT Science, Revelstoke, BC, Canada; 6 Kitman Labs, Performance Research Intelligence Initiative, Dublin, Ireland; 7 Department of Human Physiology, University of Oregon, Eugene, Oregon, United States of America; 8 Sports Performance Unit, Tasmanian Institute of Sport, Launceston, Tasmania, Australia; Universiti Malaya, MALAYSIA

## Abstract

This study investigated whether heat acclimation (HA) could improve rowing performance in temperate conditions in national-level rowers. Using a parallel-group design, eleven rowers (3 female, 8 male, age: 21±3 years, height: 182.3±6.8cm, mass: 79.2±9.0kg, $\dot{V}O_{2peak}$: 61.4±5.1ml·kg·min^-1^) completed either a HA intervention (HEAT, n = 5) or acted as controls (CON, n = 6). The intervention replaced usual cross-training sessions and consisted of an hour of submaximal cycling or rowing ergometry in either 34±0°C for HEAT or 14±1°C for CON daily over two five-day blocks (10 sessions total), separated by 72h. Participants performed the ‘10+4’ test that consists of 10-min submaximal rowing and a 4-min time-trial (TT) in temperate conditions (20±0°C) before and after the intervention. Heat acclimation following the 10-session intervention was evidenced by large significant (p<0.05) decreases in maximum tympanic temperature (d = -1.68) and rate of perceived exertion (RPE) (d = -2.26), and a large significant increase in sweat loss (d = 0.91). Large non-significant (p>0.05) decreases were seen in average tympanic temperature (d = -3.08) and average heart rate (d = -1.53) in HEAT from session 2 to session 10 of the intervention. Furthermore, a large significant increase was seen in plasma volume (d = 3.74), with large significant decreases in haemoglobin concentration (d = -1.78) and hematocrit (d = -12.9). Following the intervention, large non-significant increases in respiratory exchange ratio (d = 0.87) and blood lactate (d = 1.40) as well as a large non-significant decrease in RPE (d = -1.23) were seen in HEAT during the 10-min submaximal rowing. A large significant decrease in peak heart rate (d = -2.27), as well as a large non-significant decrease in relative $\dot{V}O_{2peak}$ (d = -0.90) and large non-significant increases in respiratory exchange ratio (d = 1.18), blood lactate concentration (d = 1.25) and power output (d = 0.96) were seen in HEAT during the 4-min TT. This study suggests that a 10-session HA intervention may elicit HA in national-level rowers, with potential to improve 4-min TT performance in temperate conditions.

## Introduction

Heat acclimation (HA) is the repeated exposure to high environmental temperatures to cause favourable physiological and perceptual adaptations that may not be achievable when exercising in cool and temperate conditions alone. Adaptions such as plasma volume (PV) expansion, lower heart rate (HR) [1], decreased rating of perceived exertion (RPE) [2] and reduced core temperature during exercise [2] are signs of HA thought to enhance exercise economy [3,4], increase maximal oxygen uptake ($\dot{V}O_{2\max}$) [5,6], and increase power at lactate threshold [5]. All of which may improve athletic performance in both hot [7,8] and cool environments [5,6,9].

Rowing athletes appear to be a cohort that could benefit from HA, as $\dot{V}O_{2\max}$ [10–12], power output at $\dot{V}O_{2\max}$ [10,11] and power output at a blood lactate concentration of 4mmol·l^-1^[10] are all favourable adaptions of HA that are highly correlated with rowing performance. However, to date, literature surrounding the effectiveness of HA on rowing performance is scarce, with to the authors’ knowledge, only one study having examined the impact of HA directly on rowing performance [8]. Garrett and colleagues [8] found that HA (90 min·d^-1^ for 5 days) caused a 4.5% plasma volume (PV) expansion measured at rest, lower HR (-14 bpm) and rectal temperature (-0.3°C) at the completion of 20 minutes of submaximal rowing, along with a quicker time to complete 2000m (-4s, -1.5%) in hot conditions (35°C). Garrett et al. [8] suggested the PV expansion led to lower HR and core temperature, and improved cardiovascular stability, during the 2000m row, which resulted in greater power output and faster times. Although the findings of Garrett et al. [8] are promising, not all rowing events are held in hot environments. For example, prestigious events such as the Oxford and Cambridge boat race and the Head of the Charles regatta are held in spring and autumn months when average maximum daily temperatures are commonly 12–19°C [13,14]. Furthermore, Australian national rowing team laboratory performance tests are completed in temperate conditions (20°C). Therefore, the effect of HA on rowing performance in cool or temperate climates warrants investigation in order to benefit well-trained rowing athletes looking to improve performance in either temperate-condition competition or selection criteria events.

The topic of HA as an ergogenic aid in cool and temperate conditions has been previously debated. Minson and Cotter [15] support the use for HA in cool and temperate conditions, suggesting physiological adaptations such as PV-induced improvements in $\dot{V}O_{2\max}$ as potential mechanisms for improvement. Minson and Cotter [15] present the findings from Lorenzo et al. [5] to illustrate the potential of HA on cool environments. Lorenzo et al. [5] showed that HA could improve $\dot{V}O_{2\max}$ and 60-min cycling time-trial performance in highly-trained cyclists in both hot (35°C) and cool environments (13°C). Nybo and Lundby [16] on the other hand, suggest that evidence for the use of HA to improve performance in cool and temperate conditions is limited, pointing to two control-matched studies [17,18] that show no relationship between PV expansion and performance and/or no significant improvement in time-trial performance in cool or temperate conditions following HA. Furthermore, Nybo and Lundby [16] suggest that the positive relationship seen between HA-induced PV expansion and performance is more likely in untrained individuals in comparison to well-trained and elite athletes, as elite athletes already possess many adaptations consistent with HA, such as increased PV, cardiac output and stroke volume [19]. Given the current debate, research that adds to the literature surrounding the effect of HA on temperate-condition performance in well-trained aerobic athletes would be of benefit.

Previous studies have found HA benefits performance in cooler environments only when exercise is prescribed at either an absolute exercise intensity during HA [5,6,20], or when HA is an additional training stimulus during [21] or after training [9], thereby increasing physiological load. This suggests an increase in training stress plays an important role in the performance improvements following HA [21,22]. Exercising in the heat is more stressful than performing the same exercise in cool or temperate environments because numerous physiological systems must function at a higher capacity to dissipate heat to avoid hyperthermia while maintaining central blood volume [23] and muscular power [24]. Therefore, a protocol that increases training load/stress in a rowers current training program may be a useful strategy for the practitioner to elicit performance gains in cool or temperate conditions. Heat acclimation is one of the only training modalities that enables an athlete to increase cardiovascular strain without a subsequent increase in mechanical load by way of increased force or power output. This has been demonstrated as both a maintenance of mechanical load (power output) with a concomitant increase in HR at 35°C compared to 13°C [5], and a maintenance of HR with 30% lower mechanical load in the heat compared to cool [25]. Given two of the most common sites of injury in rowing are the lumbar spine and ribs [26], and that rib stress [27] and lumbar spine compressive forces [28] are positively correlated to increases in rowing handle power output, thus increased mechanical load, a method such as HA that allows a relative increase in intensity without a subsequent increase in force production would be highly valuable in well-trained rowers.

The aim of this study was to investigate the effect of HA on temperate condition rowing performance in national-level rowing athletes. We hypothesised that a HA intervention consisting of regular submaximal training prescribed via absolute intensity in the heat would elicit greater physiological and perceptual adaptations during submaximal and maximal intensity rowing when compared to performed in cool-temperate conditions. Ultimately, such adaptations would lead to improved 4-min TT rowing ergometer performance in a temperate environment (20°C), whilst minimising additional mechanical stress to the athlete.

## Materials and methods

### Experimental approach to problem

**Fig 1:**
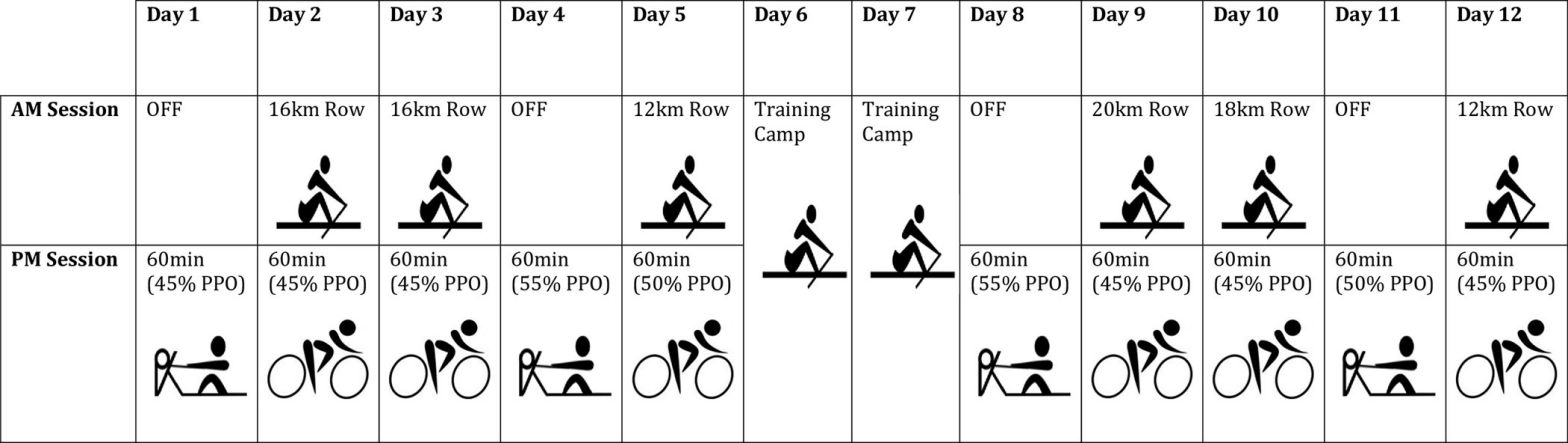
Schematic of training program during a 10-session heat acclimation intervention in national-level rowers. PPO: 4-min rowing ergometer time-trial average power output.

This investigation utilised a 10-session parallel-group study design, whereby national-level rowers were allocated to perform either rowing or cycling ergometer exercise in either 34°C (HEAT) or 15°C (CON) for 60-min. A 30-min TT was conducted before the intervention, whilst the ‘10+4’ test, the tests used by the Australian Rowing team at the time of the study [29], were conducted before (baseline) and after the intervention to determine rowing performance in temperate conditions. Physiological, perceptual and blood measures consistent with HA were collected during the intervention period. Performance (average power output), perceptual and physiological measures, including gas analysis were collected during the ‘10+4’. A schematic diagram of the intervention design can be seen in Fig 1.

### Participants

Twelve well-trained rowers were recruited for this study, of which eleven (3 female, 8 male, age: 21 ± 3 years, height: 182.3 ± 6.8cm, mass: 79.2 ± 9.0kg, $\dot{V}O_{2peak}$: 61.4 ± 5.1ml.kg.min^-1^) completed the entire intervention. Participants were allocated to a HA group [HEAT, n = 5 (4 male, 1 female), mass: 81.3 ± 6.0kg, $\dot{V}O_{2peak}$: 62.4 ± 5.2ml·kg^-1^·min^-1^, 4-min PO: 4.71W.kg^-1^] or control group [CON, n = 6 (4 male, 2 female), mass: 77.4 ± 11.2kg, $\dot{V}O_{2peak}$: 60.6 ±5.4ml·kg^-1^·min^-1^, 4-min PO: 4.70W.kg^-1^]. Groups were matched by rowing competition class (e.g., heavyweight men, heavyweight female, etc.) and 30-min time-trial (TT) performance. All subjects had competed at a national level within the past year. Due to illness, one female HEAT participant withdrew from the study. Participants provided written informed consent and the study was approved by the Institutional Human Research Ethics Committee (Ethics no. H0013872), which conformed to the recommendations of the Declaration of Helsinki.

### Procedures

#### Intervention

This study was undertaken in Tasmania during August (average maximum daily temperature was 14 ± 2°C) [30]. Athletes were in their respective state-level competitive season, approximately four weeks prior to their first national time trial selection commitment. All participants were required to continue normal on-water training sessions as prescribed by the Tasmanian Institute of Sport rowing coach. The intervention replaced regular afternoon cross-training sessions of similar duration and intensity, with all intervention sessions occurring between 3:00–6:00pm. Prescribed training can be seen in Fig 1.

The interventions consisted of two 5-day periods separated by 72h. During each 5-day period, participants either rowed or cycled on ergometers (Concept 2, Morrisville, Vermont, USA and Wattbike pro, Nottingham, UK, respectively) for 60-min/day. Exercise intensity was prescribed at 45–55% of average power output (PO_ave_) achieved in the baseline 4-min TT (‘4’), with specific absolute intensities prescribed for each session (Fig 1). HEAT exercised in an environmental chamber set to 34°C (34 ± 0°C, 55 ± 4% RH), whereas CON exercised in ambient conditions inside team rowing sheds (14 ± 1°C, 57 ± 6% RH).

Rowing sessions consisted of five 10-min blocks, each separated by 1-min of passive recovery, and a final 5-min row. Cycle sessions consisted of 60-min cycling at a constant absolute intensity relative to a corresponding rowing intensity calculated using the formula, which was developed from previous test data from our laboratories:

Cyclepower(W)=103.3(0.0112xrowingpoweroutput+0.8028)–99.8

This formula was created by regression equation between power output and submaximal $\dot{V}O_{2}$ for rowing and cycling lab testing, conducted on Tasmanian Institute of Sport athletes (unpublished data). All participants followed the intervention schedule as seen in Fig 1.

Water (2.5ml·kg^-1^ of body mass) was provided to each participant during intervention sessions with the instruction to consume it all during exercise. Body mass was measured before and after exercise in order to estimate sweat loss. It was assumed that body mass loss (g) equated to sweat loss (ml). Water intake was adjusted for when determining sweat loss. Tympanic temperature (tymp) (Thermoscan, Braun GmbH, Kronberg, Germany) was measured before exercise (before entering the heat chamber for HEAT), and every 5-min during cycling and following each 10-min interval during rowing. Tymp was the preferred means of temperature measurement in order to reduce the amount of invasion and discomfort required with other measurement strategies such as rectal or oral methods. HR was recorded (Polar, Oulu, Finland) at 5-min intervals and RPE was recorded every 10-min and immediately post exercise for both exercise modalities. RPE presented is the average of the recorded time points for each session. PO_ave_ was recorded for every 5-10-min rowing interval and every 60-min of cycling, with the final average output recorded at the end of the session presented. Physiological data was compared between Session 2 and Session 10 of the intervention in order to determine if HA occurred. Session 2 and Session 10 were utilised as this allowed comparison of the same prescribed exercise modality and intensity. Given this study was conducted within the national institute setting, with specific goals and structure of cross-training sessions, the intervention schedule was determined to be best for the overall structure of the participants training plan. The authors were confident that the changes between session 1 and session 2 would be minimal as literature suggests heat acclimation adaptations such as plasma volume expansion, HR and changes in core temperature usually takes ≥3 days to present [31], therefore, allowing an opportunity to investigate whether HA occurred whilst not imposing on the preferred training plan of the head coach.

#### Testing methods

All testing procedures were conducted in accordance with Rowing Australia national testing protocols [29]. Both groups completed a 30-min TT prior and the ‘10+4’ rowing ergometer test prior to and following the exercise intervention. The 30-min TT was performed at local rowing club sheds (in 14.0°C), after a self-regulated warm up, 9 to 10 days before the ‘10+4’. Participants performed the ‘10+4’ within 5 days either side of the intervention in a controlled 20°C laboratory. The ‘10+4’ involved participants rowing for 10-min at 95% of 30-min TT PO_ave_ (the ‘10’), resting for 5-min, completing a self-selected warm-up, then performing a 4-min TT (the ‘4’).

During the ‘10+4’, open circuit spirometry (TrueOne 2400, Parvomedics, Sandy, UT, USA) was used to determine: average $\dot{V}O_{2}$ and average respiratory exchange ratio (RER) for the final 4-min of the ‘10’, $\dot{V}O_{2peak}$ (the highest 15s average) during the ‘4’, and average RER for the final minute of the ‘4’. Carbohydrate oxidation and fat oxidation were calculated from open circuit spirometry via the following equations; CHO oxidation = (4.21 x $\dot{V}CO_{2}$)–(2.962 x $\dot{V}O_{2}$); Fat oxidation = (1.695 x $\dot{V}$)_2_)–(1.701 x $\dot{V}{\mathrm{CO}}_{2}$) [32]. HR (Polar, Oulu, Finland) was measured continuously throughout the ‘10+4’ to determine the average HR for the final 4-min of the ‘10’ and HR_peak_ (the highest measured HR) during the ‘4’. Blood lactate (BLA) was measured (Lactate Pro, Arkray KDK, Japan) before and after the ‘10’, and immediately and 2-min post the ‘4’, in finger prick samples (5μL) [33]. Maximal BLA was determined as the higher of the two post- ‘4’ measurements. Body mass (while wearing a rowing suit) was measured to ±20g before and after each ‘10+4’ to estimate sweat loss. All participants completed a familiarisation of the ‘10+4’ in a 20°C laboratory one week before baseline ‘10+4’ testing and were familiar with the 30-min TT as it was part of their regular monitoring. All testing was performed on rowing ergometers (Concept II Morrisville, Vermont, USA).

Changes in plasma volume (PV) were calculated from haematocrit (Hct) and haemoglobin concentration ([Hb]) using the mathematical equation developed by Dill and Costill [34], with previous unpublished data from our laboratories calculating a technical error of 4% for this measure. All finger prick samples (100 uL) were measured before exercise, after sitting passively for 10-min. Finger prick samples were collected 5-days prior to the intervention and then on Day 9 of the intervention for CON and day 10 for HEAT. [Hb] and Hct were determined from the average of duplicates using a HemoCue® Hb 20 (Hemocue AB, Ängelholm, Sweden) and the capillary centrifuge method (12,000 rpm for 5-min), respectively. The difference in collection days was due to unavoidable differences in the availability of CON compared to HEAT. Based on results of previous 10-day acclimation literature [5], it was assumed the one day time difference would have minimal effect of Hct and [Hb], whilst the pre-intervention measures were taken on the same day of the baseline ‘10+4’ to allow for as little interruption to the athletes schedule. It was assumed that given the level of athlete and consistency of training that minimal differences would be seen between pre- and Day 1 of the intervention.

### Statistical analyses

All data were assessed for normality of distribution using the Shapiro-Wilk test via statistical software (Graphpad, Prism 9, version 9.2.0). Data are presented as mean ± standard deviation (± SD). Data that failed the Shapiro-Wilk test for normality were log-transformed before analysis to reduce bias arising from non-uniformity in error [35]. Statistical significance was set at p<0.05, while effect size (Cohen’s d) is the predominant statistic variable used for assessing change, in line with recommendations from recent American Statistician journal editorials and American Association of Statistics position statement [36,37]. Cohen’s d (d) were calculated using the standard deviation of the mean difference for within-group changes, whilst between-group changes were calculated using the square root of average variance for between-group changes. Both between-group and within-group comparisons were calculated with 95% confidence limits [95% CL], with the following threshold values employed: <0.2 as trivial, >0.2 as small, >0.5 as moderate, >0.8 as large. Mixed-effects analyses were performed on all longitudinal data. Post-hoc testing was performed for changes between time points and independent sample t-tests were used for between-group changes. Statistical analyses were performed using Graphpad Prism 9 (version 9.2.0).

## Results

### Evidence of heat acclimation

Physiological and perceptual adaptations consistent with HA occurred in HEAT but not CON. When comparing within-group changes in HEAT from Session 2 to Session 10 of the intervention, large significant decreases were found in tymp_max_, RPE and sweat loss, with large non-significant decreases seen in average HR and tymp_ave_ (Table 1). No significant changes were seen in CON, however, small to moderate non-significant effects were seen in average HR, tymp_ave_, tymp_max_, RPE (Table 1). When changes were compared between groups, large significant decreases were seen in tymp_ave_, tymp_max_ and RPE, whilst average HR and sweat loss showed moderate and large non-significant changes, respectively, in HEAT when compared to CON. Group x time interactions and main effects for group and time can be seen in Table 1.

**Table 1:** Comparison of physiological and perceptual measures taken during Session 2 and Session 10 of a heat acclimation intervention in well-trained rowers.

	HEAT			CON				Main effects/ Interactions		
	Session 2	Session 10	Within-group change (Mean [95% CL])	Session 2	Session 10	Within-group change (Mean [95% CL])	Between-group change (Mean [95% CL])	Group (p =)	Time (p =)	Group x Time (p =)
Average Power (W)	187 ± 26	200 ± 32	12 [0; 25] d = 0.68 [0.00; 1.37] p = 0.05	175 ± 39	175 ± 38	0 [-12; 11] d = -0.11 [-3.91; 3.69] p = 0.95	13 [-10; 35] d = 1.03 [-0.79; 2.85] p = 0.19	0.40	0.14	0.12
Average HR (bpm)	149 ± 10	143 ± 9	-6 [-13; 1] d = -1.53 [-3.21; 0.15] p = 0.07	132 ± 10	130 ± 8	-2 [-8; 5] d = -0.20 [-0.57; 0.97] p = 0.82	-5 [-13; 4] d = -0.69 [-0.65; 2.03] p = 0.27	0.02	0.09	0.29
Average Tympanic Temperature (°C)	37.9 ± 0.1	37.5 ± 0.1	-0.4 [-0.7; 0.0] d = -3.08 [-6.17; 0.01] p = 0.05	36.8 ± 0.1	37.1 ± 0.2	0.3 [-0.0; 0.6] d = 0.61[-0.10; 1.31] p = 0.08	-0.6 [-1.1; -0.2] d = -1.86 [-3.23; -0.48] p = 0.02	0.001	0.73	0.02
Maximum Tympanic Temperature (°C)	38.8 ± 0.4	38.0 ± 0.3	-0.9 [-1.4; -0.4] d = -1.68 [-2.64; -0.72] p = 0.003	37.3 ± 0.6	37.6 ± 0.2	0.4 [-0.1; 0.8] d = 0.75 [-0.21: 1.71] p = 0.11	-1.2 [-1.9; -0.5] d = -2.49 [-3.89; -1.09] p = 0.003	0.0002	0.12	0.003
RPE (AU)	13 ± 1	11 ± 1	-2 [-3; -1] d = -2.26 [-3.76; -0.76] p = 0.01	9 ± 1	10 ± 1	0 [-1; 2] d = 0.27 [-0.49; 1.03] p = 0.45	-3 [-4; -1] d = -1.87 [-3.20; -0.54] p = 0.01	0.002	0.08	0.01
Sweat loss (L)	1.3 ± 0.3	1.6 ± 0.6	0.3 [0.0; 0.6] d = 0.91 [0.11; 1.71] p = 0.03	0.7 ± 0.3	0.6 ± 0.3	-0.0 [-0.3; 0.2] d = -0.10 [-1.38; 1.18] p = 0.86	0.4 [-0.1; 0.8] d = 1.30 [-0.22–2.82] p = 0.08	0.06	0.11	0.07

Note: Values are mean ± SD. 95% CL: 95% confidence limit. HR: Heart rate. bpm: Beats per minute. RPE: Rate of perceived exertion. AU: Arbitrary units.

**Fig 2:**
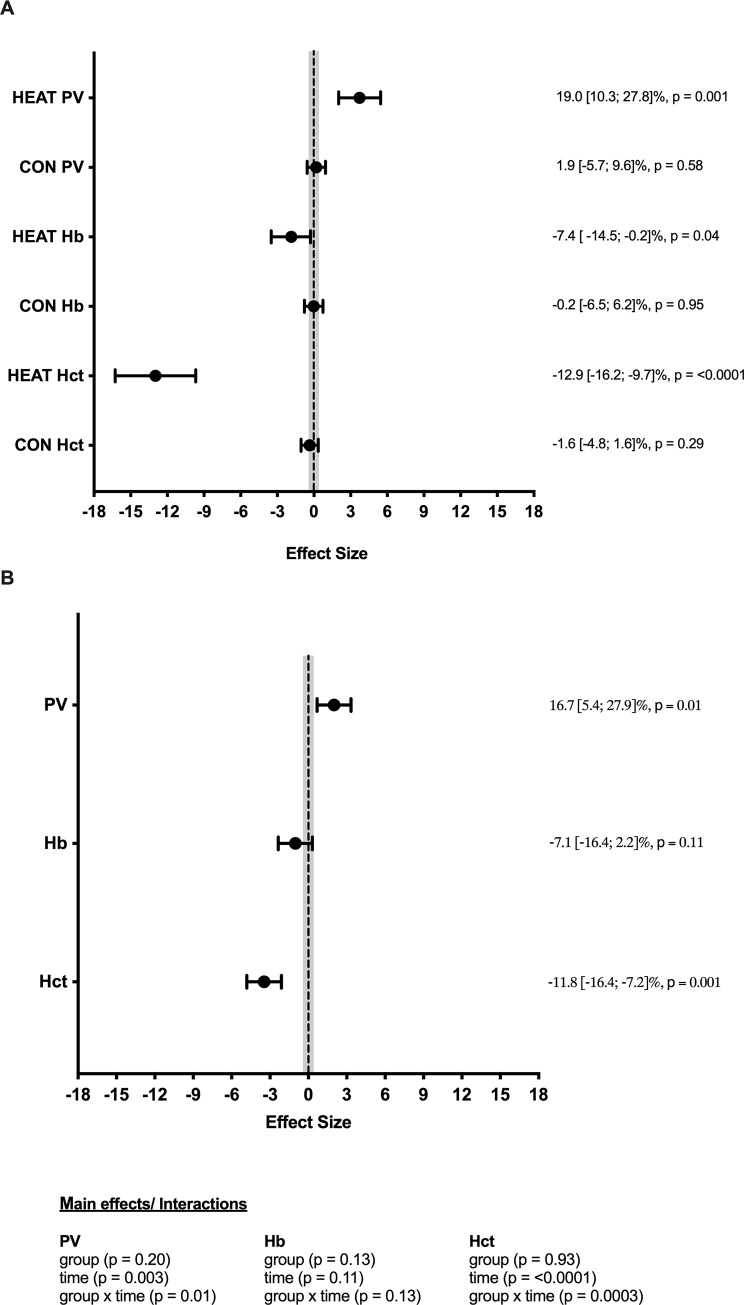
Within-group (A) and between-group (B) comparison of change in blood parameters pre–post a 10-day heat acclimation protocol in national-level athletes. Shaded area represents trivial change. Percentage change and p-values are reported on the right-hand side for each variable. Main effects and interaction are reported below.

HEAT showed a large significant increase in PV, as well as large significant decreases in Hct and [Hb] (Fig 2A). No significant changes in blood adaptations were seen in CON, with trivial changes seen in PV and [Hb], and a small non-significant decrease seen in Hct. When changes were compared between groups, there was a large significant increase in PV, a large significant decrease in Hct, as well as a large non-significant decrease in [Hb] in HEAT when compared to CON (Fig 2). Group, time and group x time interactions can be seen in Fig 2(B).

### 10-min submaximal rowing test

When changes were compared within-group from pre- to post-intervention in HEAT, a large significant increase was seen in power output, with trivial to large non-significant changes seen for all other variables. A large significant decrease in BLA was seen in CON, with trivial to large non-significant changes seen for all other variables. No significant changes were seen when comparing changes between groups pre- to post-intervention, with small to large non-significant differences seen across all variables. Group x time interactions and main effects for group and time can be seen in Table 2.

**Table 2:** Comparison of physiological and perceptual measures of national-level rowing athletes taken during a 10-min submaximal rowing test performed at 95% of 30-min time-trial average power output, before (Pre) and after (Post) a 10-session heat acclimation intervention.

	HEAT			CON				Main effects/ Interactions		
	Pre	Post	Within-group difference (Mean [95% CL])	Pre	Post	Within-group difference (Mean [95% CL])	Between-group differences (Mean [95% CL])	Group (p =)	Time (p =)	Group x Time (p =)
Average Power (W)	277 ± 37	279 ± 39	2 [0; 4] d = 0.83 [0.14; 1.52] p = 0.02	265 ± 53	265 ± 53	0 [-2; 2] d = 0.00 [-0.80; 0.80] p = 1.00	2 [-1; 6] d = 1.21 [-0.51; 3.00] p = 0.14	0.66	0.08	0.08
HR (bpm)	171 ± 10	169 ± 8	-2 [-7; 3] d = -0.49 [-1.62; 0.63] p = 0.35	183 ± 9	184 ± 8	1 [-3; 6] d = 0.23 [-0.63; 1.09] p = 0.57	-3 [-10; 3] d = -0.69 [-2.03; 0.66] p = 0.28	0.03	0.75	0.29
$\dot{V}O_{2}$ (ml·kg^-1^·min^-1^)	52.5 ± 5.1	53.0 ± 4.0	0.5 [-1.2; 2.1] d = 0.23 [-0.59; 1.05] p = 0.54	54.3 ± 3.2	54.2 ± 3.5	-0.2 [-1.7; 1.3] d = -0.15 [-1.31; 1.01] p = 0.78	0.6 [-1.8; 3.1] d = 0.39 [-1.14; 1.92] p = 0.56	0.53	0.79	0.52
RER	0.96 ± 0.03	0.98 ± 0.03	0.03 [-0.01; 0.06] d = 0.73 [-0.30; 1.76] p = 0.06	0.94 ± 0.05	0.94 ± 0.03	-0.01 [-0.04; 0.03] d = -0.15 [-1.07–0.77] p = 0.52	0.03 [-0.02; 0.08] d = 0.87 [-0.50; 2.24] p = 0.18	0.16	0.37	0.18
RPE (AU)	15 ± 1	14 ± 1	-1 [-3; 1] d = -0.43 [-1.13; 0.28] p = 0.22	13 ± 2	15 ± 1	2 [0; 3] d = 0.85 [-0.01; 1.71] p = 0.052	-3 [-6; 1] d = -1.23 [-2.68; 0.23] p = 0.09	0.29	0.65	0.03
BLA (mmol·L^-1^)	5.9 ± 2.0	5.9 ± 2.3	0.0 [-1.4; 1.4] d = 0.00 [-0.79; 0.79] p = 1.00	7.3 ± 1.8	5.4 ± 1.2	-1.9 [-3.4; -0.5] d = -1.15 [-2.00; -0.30] p = 0.01	1.9 [-0.2; 4.1] d = 1.40 [-0.12; 2.92] p = 0.07	0.69	0.06	0.06
CHO oxidation (g·L^-1^)	4.6 ± 1.0	5.1 ± 0.9	0.5 [-0.2; 1.1] d = 0.68 [-0.32; 1.67] p = 0.15	4.2 ± 1.3	4.1 ± 1.1	-0.1 [-0.7; 0.5] d = -0.19 [-1.12; 0.75] p = 0.66	0.6 [-0.3; 1.5] d = 0.66 [-0.34; 1.67] p = 0.17	0.33	0.43	0.18
Fat oxidation (g·L^-1^)	0.3 ± 0.1	0.2 ± 0.1	-0.1 [-0.3; 0.1] d = -0.64 [-1.88; 0.60] p = 0.47	0.4 ± 0.4	0.4 ± 0.2	0.0 [-0.2; 0.2] d = 0.13 [-0.72; 0.98] p = 0.93	-0.2 [-0.5; 0.2] d = -0.66 [-1.99; 0.67] p = 0.29	0.17	0.54	0.30

Note: Values are mean ± SD. 95% CL: 95% confidence limits. HR: Heart rate. bpm: Beats per minute. $\dot{V}O_{2}$: Oxygen uptake. RER: Respiratory exchange ratio. RPE: Rate of perceived exertion. BLA: Blood lactate. CHO oxidation: Carbohydrate oxidation. AU: Arbitrary units.

### 4-min Max TT

**Fig 3:**
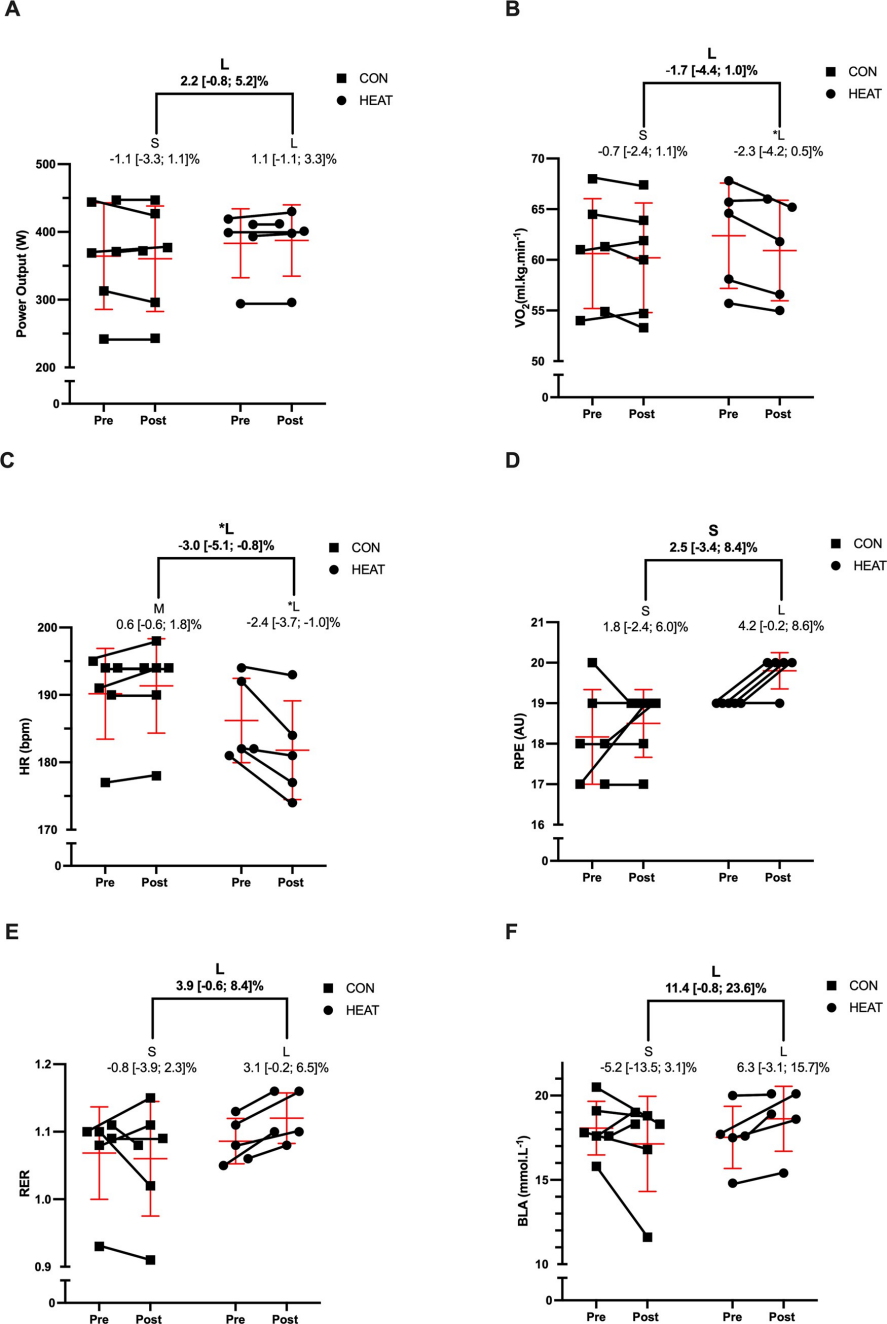
Percent change in average power output (A) relative peak oxygen uptake; $\dot{V}O_{2peak}$ (B), peak heart rate; HR_peak_ (C), rating of perceived exertion; RPE (D), respiratory exchange ratio; RER (E) and blood lactate; BLA (F) pre–post a 10-day heat acclimation in national-level rowers. Group data are presented as mean ± SD. Statistical significance (p >0.05) is denoted by the symbol (*). Effects are illustrated via letters, with the following used to identify the magnitude of change; S (small), M (medium), L (large). Bolded letters and numerals represent between-group changes whilst non-bolded represent within-group changes.

There were small non-significant differences between groups in both 4-min TT PO (d = 0.28 [-0.99; 1.55], p = 0.65) and $\dot{V}O_{2peak}$ (d = 0.33 [-0.93; 1.59], p = 0.59) at the start of the intervention. When changes were compared within-group from pre- to post-intervention in HEAT, large significant changes were seen in HR_peak_ and relative $\dot{V}O_{2peak}$, with large non-significant changes seen in average power output, RER, RPE, and BLA (Table 3). No significant changes were seen from pre- to post-intervention in CON, with small to large non-significant effects seen across the six variables measured (Table 3). When differences in change were compared between groups, HEAT showed a large significant decrease in HR_peak_, whilst average power, relative $\dot{V}O_{2peak}$, RER and BLA all showed large non-significant changes when compared to CON (Table 3). Group x time interactions and main effects for group and time can be seen in Table 3. Individual changes can be seen for all 4-min variables in Fig 3.

**Table 3:** Comparison of physiological and perceptual measures of national-level rowing athletes taken during a 4-min time trial, before (Pre) and after (Post) a 10-session heat acclimation intervention.

	HEAT				CON			Main effects/ Interactions		
	Pre	Post	Within-group differences (Mean [95% CL])	Pre	Post	Within-group differences (Mean [95% CL])	Between-group differences (Mean [95% CL])	Group (p =)	Time (p =)	Group x Time (p =)
Average Power (W)	383 ± 51	387 ± 53	4 [-4; 13] d = 0.83 [-0.87; 2.52] p = 0.30	364 ± 79	360 ± 78	-4 [-12; 4] d = -0.38 [-1.13; 0.37] p = 0.28	8 [-3; 20] d = 0.96 [-0.36; 2.29] p = 0.13	0.57	0.97	0.15
HR_peak_ (bpm)	186 ± 6	182 ± 7	-4 [-7; -2] d = -1.34 [-2.09; -0.58] p = 0.003	190 ± 7	191 ± 7	1 [-1; 3] d = 0.79 [-0.74; 2.32] p = 0.27	-6 [-10; -2] d = -2.27 [-3.91; -0.64] p = 0.02	0.13	0.06	0.01
Relative $\dot{V}O_{2}$ (ml·kg^-1^·min^-1^)	62.4 ± 5.2	60.9 ± 5.0	-1.5 [-2.6; -0.3] d = -1.12 [-2.02; -0.22] p = 0.02	60.6 ± 5.5	60.2 ± 5.4	-0.4 [-1.5; 0.7] d = -0.41 [-1.46; 0.64] p = 0.40	-1.0 [-2.7; 0.6] d = -0.90 [-2.34; 0.54] p = 0.19	0.71	0.03	0.17
Absolute $\dot{V}O_{2}$ (L·min^-1^)	5.1 ± 0.6	4.9 ± 0.6	-0.14 [-0.23.; -0.04] d = -1.53 [-2.61; -0.45] p = 0.01	4.7 ± 1.0	4.7 ± 0.9	-0.07 [-0.16; 0.02] d = -0.70 [-1.58; 0.18] p = 0.11	-0.07 [-0.20; 0.06] d = -0.69 [-2.05; 0.66] p = 0.28	0.53	0.006	0.28
RER	1.09 ± 0.03	1.12 ± 0.04	0.03 [0.00; 0.07] d = 2.26 [-0.21; 4.73] p = 0.06	1.07 ± 0.07	1.06 ± 0.09	-0.01 [-0.04; 0.03] d = -0.20 [-0.92; 0.52] p = 0.59	0.04 [-0.01; 0.09] d = 1.18 [-0.17; 2.53] p = 0.08	0.31	0.27	0.08
RPE (AU)	19 ± 0	20 ± 1	1 [0; 2] d = 1.79 [-0.08; 3.66] p = 0.06	18 ± 1	19 ± 1	0 [0; 1] d = 0.32 [-0.42; 1.06] p = 0.35	1 [-2; 4] d = 0.49 [-0.67; 1.65] p = 0.35	0.03	0.0496	0.38
BLA (mmol·L^-1^)	17.5 ± 1.8	18.6 ± 1.9	[-0.5; 2.7] d = 1.27 [-0.63; 3.18]p = 0.17	18.1 ± 1.6	17.1 ± 2.8	-0.9 [-2.4; 0.6] d = -0.46 [-1.19; 0.28] p = 0.19	2.0 [-0.1; 4.2] d = 1.25 [-0.09; 2.59] p = 0.06	0.70	0.87	0.07

Note: Values are mean ± SD. 95% CL: 95% confidence limits. HR: Heart rate. Bpm: Beats per minute. $\dot{V}O_{2}$: Oxygen uptake. RER: Respiratory exchange ratio. RPE: Rate of perceived exertion. BLA: Blood lactate.

## Discussion

The aim of the current study was to firstly investigate if a 10-session HA protocol could induce favourable physiological and perceptual adaptations during submaximal and maximal rowing. Secondly, if these adaptations ultimately improve 4-min TT performance within temperate conditions in national-level rowers. Following a 10-session (60-min/session) HA protocol, HEAT elicited numerous physiological adaptations consistent with HA, including large changes in blood parameters such as PV, Hb and Hct, along with increased sweat loss, decreased tympanic temperature and decreased HR at a prescribed absolute intensity in hot conditions. When comparing HEAT to CON during the ‘10+4 rowing ergometer test in temperate conditions (20°C), HEAT showed large non-significant increases in RER and BLA, and a large non-significant decrease in RPE when rowing at 95% of 30min TT PO. When HEAT and CON were compared during the 4-min TT component of the ‘10+4’, HEAT showed a large significant decrease in HR_peak_, large non-significant decreases in RPE and $\dot{V}O_{2peak}$ and large non-significant increases in RER and PO. The findings of this study suggest that a 10-session HA exposure can enhance physiological and perceptual training adaptations in national-level rowers and, although not reaching statistical significance, HA may have the potential to improve 4-min TT performance.

Debate continues as to whether HA can improve exercise performance in temperate conditions. Opposing views on this topic were put forward in a 2016 cross talk debate, with Minson and Cotter [15] arguing in favour of HA, whilst Nybo and Lundby [16] proposed not enough evidence was available to support the use of HA to improve performance in temperate conditions. Since this debate, further evidence has arisen for both sides of the argument. McCleave et al. [20] and Rendell et al. [38] suggest that HA can improve temperate-condition TT performance in well-trained endurance athletes, whilst Neal et. al [39] found HA to improve lactate threshold but not TT performance in well-trained cyclists. Furthermore, Zurawlew et. al [40] found similar findings to those of Keiser [17] and Karlsen [18]; that despite HA, increased TT performance was seen in hot but not temperate conditions. The current study further adds to literature pertaining to the impact of HA on exercise performance, with our findings suggesting that a 10-session HA protocol can elicit many signs of HA and has the potential to improve short-duration (4-min) rowing TT performance in temperate conditions (20°C).

The large increase (d = 0.96) in 4-min PO in HEAT when compared to CON in the current study supports the use of HA as a potential ergogenic aid for exercise performance in temperate conditions, specifically short-duration rowing TT performance. Although not statistically significant (p = 0.13), performance effects of HA ranged from -3W to 20W. Given that a division of 3 has been suggested when converting rowing ergometer power improvement to seconds [41], the effects of HA on performance when comparing HEAT to CON ranged from ~1s slower through to ~7s faster in our study. As the first four positions of the 2021 Olympic men’s heavyweight Single Scull A Final were separated by no more than 2.3s in total (first place; 6:40.45, fourth place; 6:42.73) [42], the potential performance benefits of HA appear to outweigh the negatives from a practitioner perspective. It is worth highlighting that an improvement in rowing ergometer performance does not necessarily mean that an on-water improvement will be seen, as other factors such as technique, conditions and body mass may all effect on-water performance. However, significant (p<0.05) correlations have previously been shown between rowing ergometer performance and world ranking in elite-level rowers; heavyweight men (r = 0.72), lightweight men (r = 0.78), heavyweight female (r = 0.75) and lightweight female (r = 0.68) [43]. Furthermore, Nevill et al. [44] has shown a significant relationship exists between rowing ergometer performance and on-water time (r = 0.54), with even stronger associations when bodyweight is accounted for (r = 0.77). The combination of both Mikulić et al. [43] and Nevill et al.’s [44] findings suggest rowing ergometer improvements are important and reflective of the athletes on-water capability, with our findings highlighting HA as a potential ergogenic aid to improve rowing ergometer performance.

The only other study [8] to investigate the effect of HA on rowing ergometer performance was performed in hot conditions (35°C). Garrett and colleagues [8] found a 4s improvement in 2000m rowing performance in elite-level rowers following 5 x 90-min heat exposures. When converted to average power output [41], this improvement would equate to approximately 12W. In comparison, the improvement seen in our study in HEAT when compared to CON was 8W. The improvements in rowing performance seen in the Garrett et al. study [8] were accompanied by a 14 bpm decrease in HR at the completion of a 20-min submaximal rowing test, and 4.5% increase in PV from pre- to post intervention, with Garrett et al. [8] suggesting that PV expansion assisted the 2000m TT improvement seen in their study.

One of the proposed mechanisms for improved performance in temperate conditions following HA is a PV expansion associated increased $\dot{V}O_{2\max}$. In our study, HA induced a PV expansion of 19% in HEAT, while [Hb] and Hct fell by 7% and 13%, respectively. Given $\dot{V}O_{2\max}$ improvements are likely dependant on the interaction between PV expansion and [Hb], at some point PV expansion and the consequent hemodilution can be large enough that O_2_ carrying capacity is reduced [45]. For example, Coyle et al. [45] found that a 7–10% PV expansion elicited only moderate hemodilution (4%) and increased $\dot{V}O_{2\max}$ by 4%. Whereas, a PV expansion of ~17% elicited much greater hemodilution (11%) and did not improve $\dot{V}O_{2\max}$ [45]. Therefore, the 1.5 ml.kg^-1^.min^-1^ decrease in $\dot{V}O_{2peak}$ (d = -1.12) seen in HEAT in our study could potentially be explained by the 19% PV expansion (d = 3.74) and 1.1 g.L^-1^ hemodilution (d = -1.78) observed following the intervention. As rowing TT performance is highly correlated with $\dot{V}O_{2\max}$ [10–12], the 1.0 ml.kg^-1^.min^-1^ decrease in $\dot{V}O_{2peak}$ in HEAT when compared to CON (d = -0.90) theoretically has the potential to negatively impact rowing performance. Interestingly, the non-significant decrease in $\dot{V}O_{2peak}$ was accompanied by a large significant decrease in HR_peak_. This decrease in HR_peak_ may be a result of the large PV expansion seen in the HEAT, theoretically leading to an increase in stroke volume and a subsequent maintenance of cardiac output with a decreased HR. However, as both stroke volume and cardiac output were not measured during our study, further research is required to substantiate this proposed mechanism. Nevertheless, the large significant and non-significant decreases in HR_peak_ and $\dot{V}O_{2peak}$, respectively, did not appear to negatively affect 4-min PO in the national-level rowers in our study, with HEAT showing potentially large improvements in rowing performance. Therefore, potential improvements in 4-min PO seen following HA in our study may possibly be explained by physiological and perceptual variables other than $\dot{V}O_{2peak}$.

Further possible explanations for the improvement seen in 4-min TT performance in HEAT during our study include greater anaerobic energy utilisation during the ‘10 + 4’ or the greater relative intensity performed by HEAT during the intervention sessions. The large increase in RER and BLA during the 4-min TT combined with the moderate increase and decrease in carbohydrate oxidation and fat oxidation respectively during the 10-min submaximal work effort, suggest that an increased utilisation of the anaerobic energy system may have occurred. Given anerobic energy system contribution has been shown to account for approximately 21–30% of energy supply during 2000m rowing ergometer TT’s [46] and as much as 50–60% in 1000m rowing ergometer TT’s [47], greater anaerobic energy utilisation has the potential to improve performance in short-duration all-out efforts. As the distance rowed during the 4-min TT in this study would sit somewhere between these two distances (1000-2000m), an increased ability to produce high levels of anerobic glycolysis could potentially account for the large improvements seen in HEAT when compared to CON. However, the fact that HEAT exercised at ~10% greater relative intensity than CON during the intervention cannot be disregarded and may have contributed to the greater 4-min TT performance seen. While possible, it would seem unlikely that a ~10% greater relative intensity over a 10-day period would have a large effect on national-level athletes TT performance due to the athletes already high levels of aerobic fitness. Nevertheless, as neither the measurement of energy system contribution nor the effect of relative versus absolute intensity prescription were primary aims of the study, coupled with dietary intake not stringently controlled during the 24h preceding performance testing, further research into these areas would be worthwhile.

Despite the promising results of the present study, the authors acknowledge the small sample size used in this study as a limitation. This was due to the limited availability of national-level rowers, which were selected for this study to ensure our findings were applicable to high-level athletes. Therefore, future HA rowing studies involving greater participants to allow clearer interpretations of findings would be of significant value. The authors also acknowledge the limitations of tympanic temperature, with opposing findings regarding the efficacy of tympanic temperature to determine change in core temperature [48–52]. For example, Easton et al. [50] and Huggins et al. [53] have shown tympanic temperature to underestimate core temperature during exercise by 0.9–1.1°C. In contrast, a recent study by Fenemor et al. [48] showed no significant difference between core temperature recordings assessed via tympanic temperature or ingestible core temperature pills, with a mean bias of 0.1°C and a CV of 1.0%. Given this conflicting evidence, the authors suggest that caution should be taken when using tympanic temperature to determine changes in core temperature. However, tympanic temperature measurements may provide an indication of temperature change within the athletes in a less invasive method than either rectal or ingestible core temperature pills. The authors also recommend that tympanic temperature should not be used in isolation to ascertain whether heat acclimation occurred, but its use in conjunction with other physiological and perceptual measures such as HR, RPE and sweat loss may assist with such inferences.

In conclusion, our study adds a novel perspective on the effectiveness of HA to improve rowing performance in temperate conditions. Our study shows that a 10-session HA protocol may elicit HA and has the potential to improve temperate-condition 4-min TT performance in national-level rowers.
